# Fishbone Foreign Body Ingestion With Gastric Impaction and Intestinal Micro-perforation in an Adult Female: A Case Report

**DOI:** 10.7759/cureus.72099

**Published:** 2024-10-22

**Authors:** David H Dupont, Louis Maria Umejiego, Savni Satoskar, NFN Soumya, Marcos Rosa Santana, Anshuman Sikka, Nitsuh Ayele, Cesar Riera Gonzalez, Nivard Bahadur, Ajit Singh

**Affiliations:** 1 General Surgery, St. George's University School of Medicine, New York, USA; 2 General Surgery, American University of the Caribbean School of Medicine, Detroit, USA; 3 General Surgery, BronxCare Health System, Bronx, USA

**Keywords:** fishbone, fishbone foreign body ingestion, fishbone impaction, fishbone injury, foreign body ingestion in adults, gastroenterology and endoscopy, lower gastrointestinal tract, micro-perforation, surgical case report, upper gastrointestinal tract

## Abstract

Foreign body ingestion of fishbones is a very common complaint where most foreign bodies travel safely through the gastrointestinal tract (GIT) without any serious complications. However, its clinical presentation is nonspecific, and its clinical severity can vary widely, thus requiring the use of conservative and or invasive treatment modalities. In this case report, we present a case of a 42-year-old female who reported eating fish two days prior to presenting with upper gastrointestinal tract (GIT) foreign body impaction in addition to a lower GIT micro-perforation secondary to fishbone ingestion, both of which were successfully managed with conservative, nonsurgical treatment modalities. Impaction, perforation, or obstruction of fishbone foreign bodies often occur at GIT angulations or narrowing. Clinical diagnosis of foreign body ingestion requires the use of multiple modalities such as a detailed history, physical exam, radiographic evaluation, and endoscopic evaluation as needed. Treatment depends on multiple factors and can be conservative or surgical in nature. Fishbone foreign body ingestion is a common complaint and rarely leads to severe complications. However, its diagnosis can be difficult without an explicit history highlighting ingestion of fishbones and requires the use of appropriate imaging modalities such as computed tomography (CT) scans. Subsequent management may require conservative or invasive treatment modalities based on the location of the fishbone, and the presence or absence of accompanying complications such as peritoneal signs, sepsis, and radiographic identification of bowel perforation.

## Introduction

Fishbone ingestion is common and accounts for approximately 48%-88% of ingested foreign bodies [1]. Pre-endoscopic series have shown that 80% or more of foreign objects will likely pass without the need for intervention [2]. However, in approximately 1% of fishbone foreign body ingestion cases, fishbones may perforate the gastrointestinal tract (GIT), especially near the terminal ileum while large bowel perforation is extremely uncommon [1].

Most of the available literature and guidelines surrounding foreign body ingestion focus on the management of upper GIT foreign body impaction and surgical management of the secondary complications that can arise from foreign body perforations of the lower GIT.

In this case report, we present a case where a 42-year-old female presented with upper GIT foreign body impaction in addition to a lower GIT micro-perforation secondary to fishbone ingestion, both of which were successfully managed with conservative, nonsurgical treatment modalities.

## Case presentation

A 42-year-old female patient with no significant past medical or surgical history presented to the Emergency Department (ED) with complaints of worsening abdominal pain, nausea, and one episode of emesis. The only inciting factor that the patient remembered was the consumption of fish, tilapia, two days prior.

**Figure 1:**
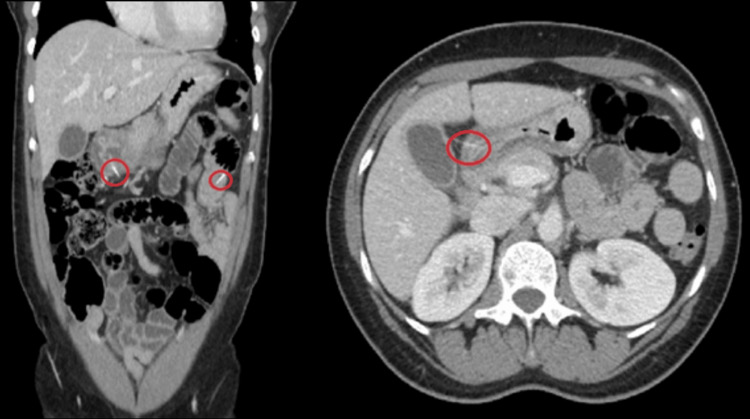
Initial CT scan showing gastric and jejunal foreign bodies

**Figure 2:**
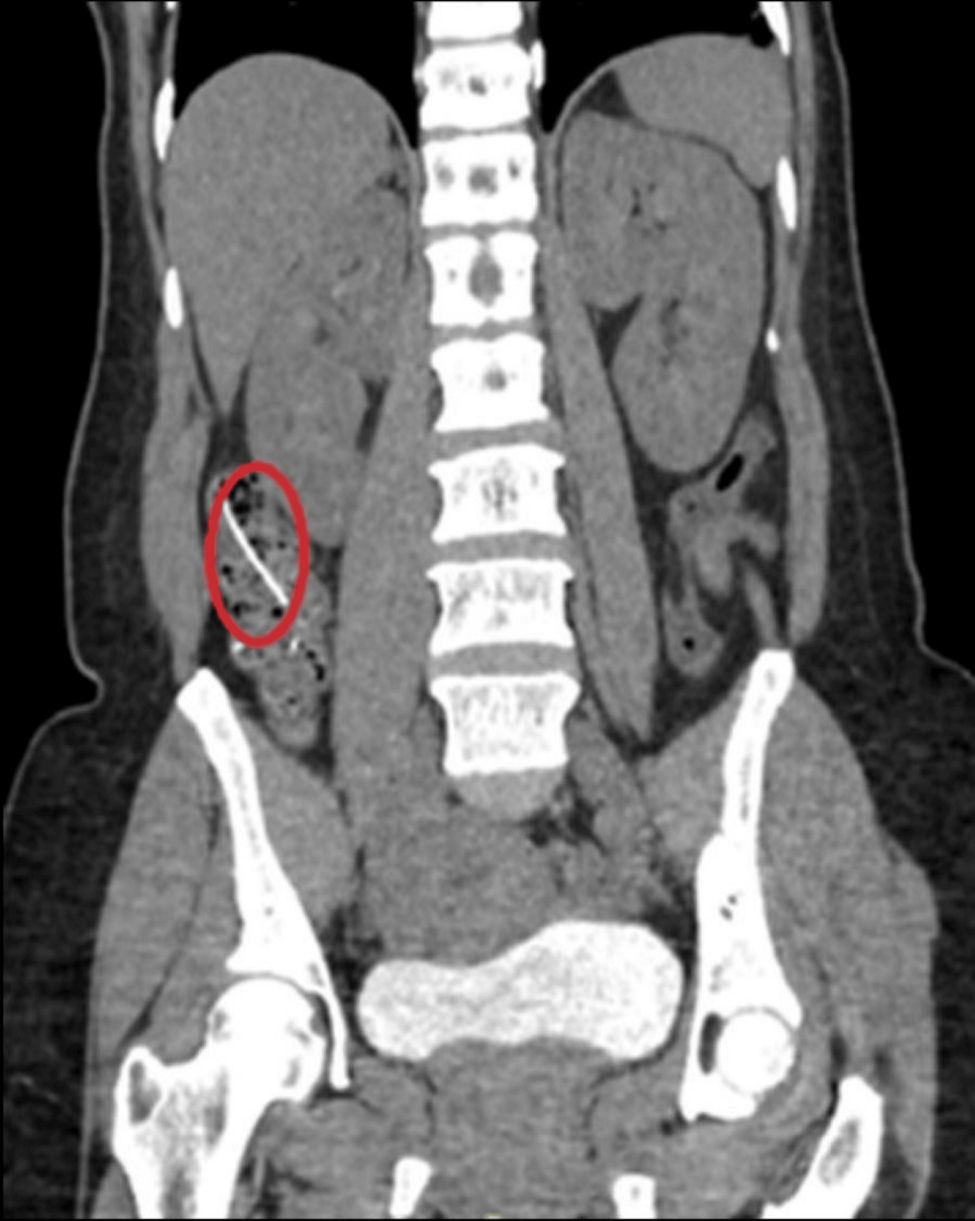
Repeat CT scan on admission day 2 showing a foreign body in the ascending colon

On examination, the abdomen was soft, tender in the epigastric region, left lower quadrant, and right lower quadrant, nondistended, without rebound, guarding, rigidity, or peritoneal signs. On initial workup, the WBC was 12.6K and omputed tomography (CT) scan showed linear densities in the stomach, jejunum, and right colon, with focal wall thickening of the gastric antrum and linear densities extending through the thickened gastric wall, possible foreign body perforation, possibly by a fishbone. CT showed no signs of pneumoperitoneum or extraluminal fluid (Figures 1-2).

**Figure 3:**
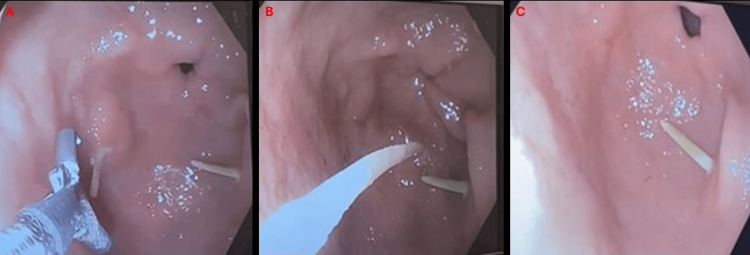
Esophagogastroduodenoscopy demonstrating impaction of two fishbones in the stomach, at the greater curvature and the gastric antrum. Esophagogastroduodenoscopy (EGD) demonstrating (A) impaction of two fishbones in the stomach. (B) One fishbone at the greater curvature. (C) One fishbone at the gastric antrum

**Figure 4:**
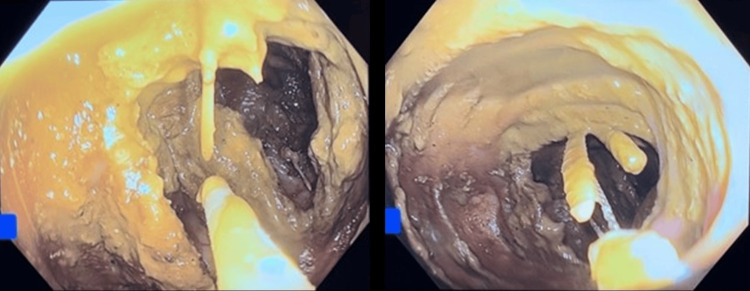
Colonoscopy images demonstrating impaction of multiple fishbones within the ascending colon

**Figure 5:**
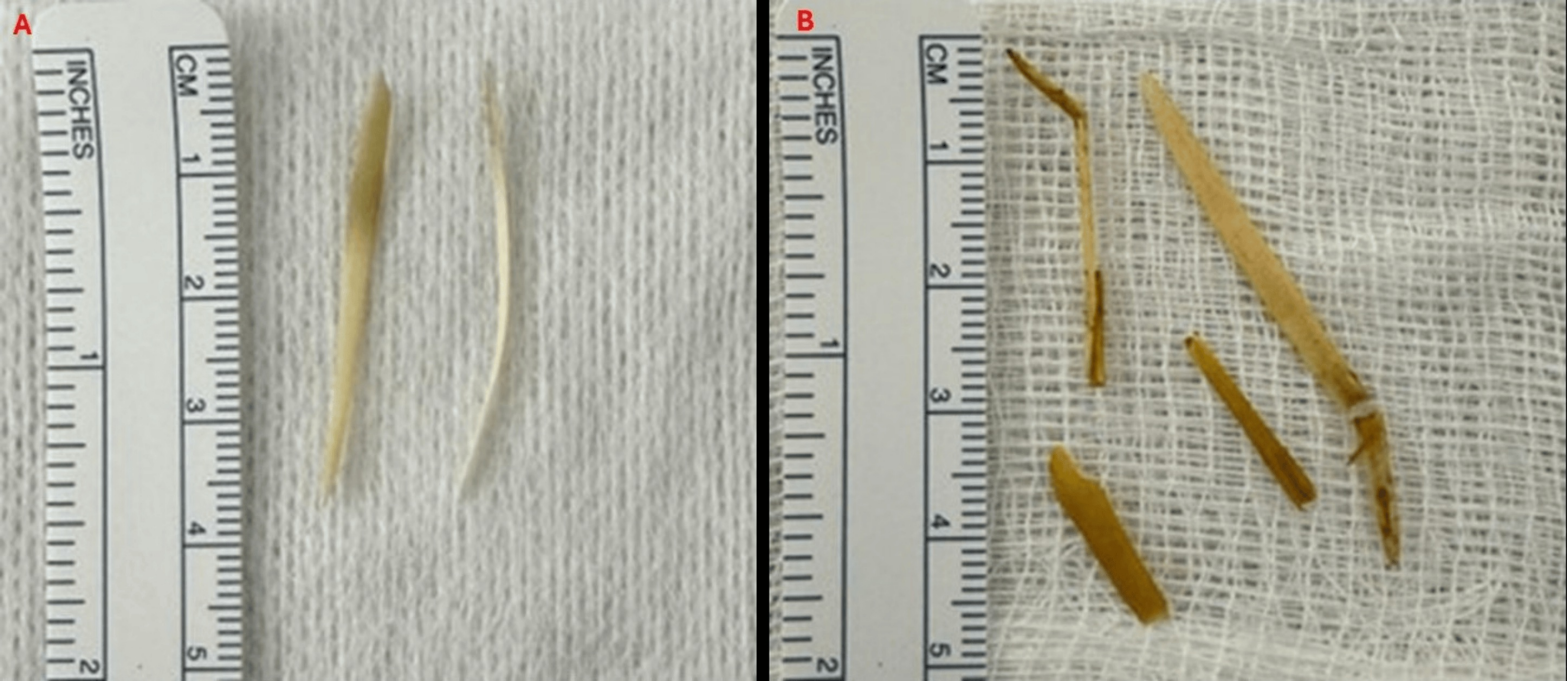
Fishbone foreign bodies removed from the stomach and colon Fishbone foreign bodies removed from the stomach (A) and colon (B), respectively

Gastroenterology (GI) was consulted for endoscopic intervention, which GI deemed not recommended at that time. On the second day of admission, after further discussion, the patient was scheduled for emergent esophagogastroduodenoscopy (EGD) and colonoscopy in the GI suite with the presence of the surgical team at the bedside. EGD was done with the latex hood and Raptor grasping device under minimal insufflation. EGD was then followed by colonoscopy. Patient tolerated both procedures well. EGD findings included one fishbone at the greater curvature of the gastric antrum, one fishbone in the lesser curvature of the gastric antrum with no visible defects, perforation, or bleeding. During the colonoscopy five fishbones were removed with a snare from the ascending colon (Figures 3-5).

**Figure 6:**
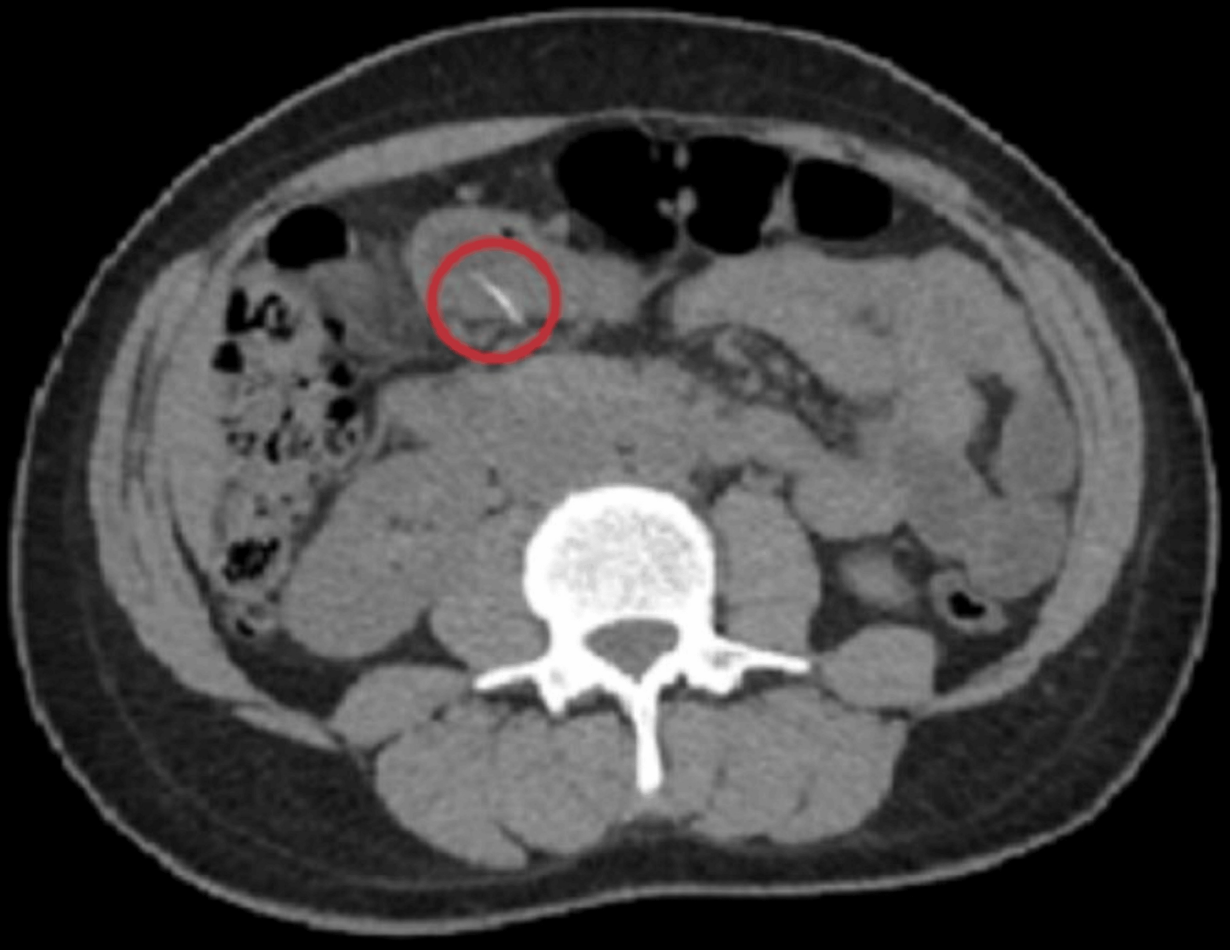
Jejunal fishbone now entirely displaced into the mesenteric fat

Repeat CT anteroposterior (A/P) on the day of EGD/colonoscopy showed that the thin linear radiodensity previously seen within the jejunum is now seen entirely within the mesenteric fat, with no accompanying inflammatory changes or evidence of pneumoperitoneum (Figure 6).

However, while repeat CT showed that the foreign body was no longer in the small bowel lumen but in the mesentery, the patient remained stable with no worsening in symptoms or signs of an acute abdomen. The patient was managed conservatively with bowel rest, antibiotic treatment, gastrointestinal prophylaxis, among other measures. The presumptive diagnosis was that of probable micro-perforation of the jejunum by the fishbone in the left upper quadrant (LUQ) during its movement into the mesentery, with no accompanying pneumoperitoneum suggesting an absence of free perforation into the peritoneal cavity. Early operation was avoided due to the possibility of the fishbone being displaced by surgical intervention if attempted, thus increasing the risk of further bowel injury during surgery, in a patient whose symptoms were not severe or worsening with initial nonoperative treatment.

The patient had complaints of mild abdominal pain on the left side of the abdomen, moving from the LUQ to the left lower quadrant with the passage of days. The patient had WBC counts within normal limits, no signs of peritonitis or gastrointestinal disturbances during her inpatient admission. Abdominal pain resolved by the fourth day of admission, with return of full bowel function and capacity to tolerate feedings. The patient was discharged from the hospital in safe condition to home on ciprofloxacin and metronidazole.

Three days after hospital discharge, the patient presented to the emergency department with complaints of abdominal cramping, nausea, vomiting, diarrhea, and dry cough with normal WBC count. She was being worked up for possible acute gastroenteritis but elected to pursue a self-directed discharge from the emergency department.

One week after her initial hospital discharge, she was seen in the surgery clinic and only reported intermittent abdominal pain and a benign abdomen on examination.

**Figure 7:**
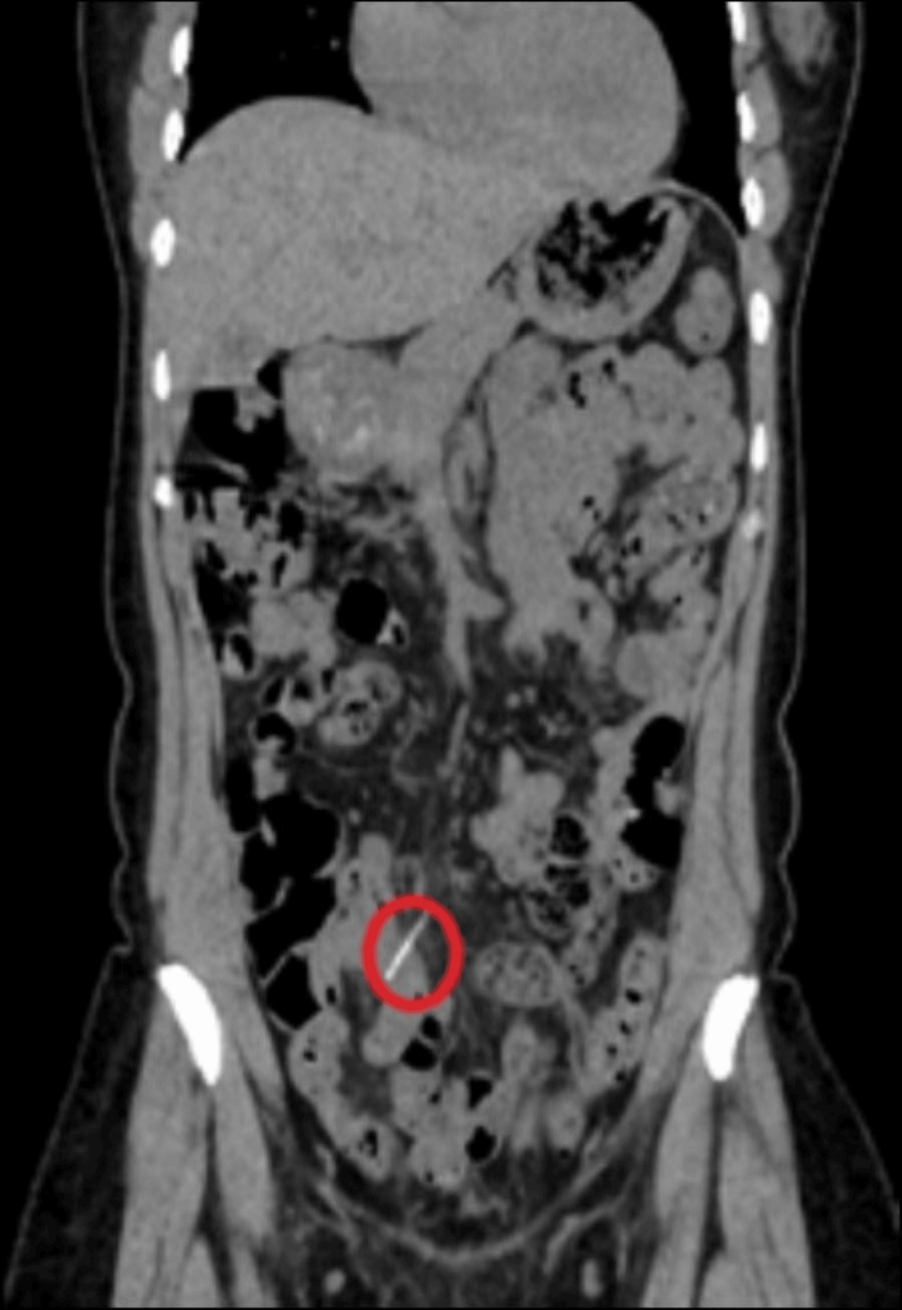
A 3.5 cm retained fishbone within the distal small bowel in the RLQ extending to the adjacent mesenteric fat RLQ: Right lower quadrant

She was readmitted 10 days after hospital discharge due to intermittent right lower quadrant abdominal pain. The patient underwent new imaging with CT A/P showing a 3.5 cm linear foreign body within a distal small bowel in the right lower quadrant extending to the adjacent mesenteric fat. There was no evidence of obstruction or abscess formation. The patient was admitted for symptom management, and her laboratory results were within normal limits throughout this admission. The patient was discharged two days later after improvement of initial symptoms (Figure 7).

On day 17, she was seen at the surgery clinic, where the patient complained of intermittent left-sided colicky abdominal pain, reflux, and bloating at night. The patient continues to take ibuprofen, acetaminophen, and omeprazole. The patient was tolerating a regular diet and taking stool softeners for bowel movements. The patient also complained of reddish urine and burning sensation during voiding. She denied having a fever, nausea, vomiting, or diarrhea. She was advised to stop taking nonsteroidal anti-inflammatory drugs (NSAIDs) and was referred to urology for a possible urinary tract infection.

At the one-month mark, the patient was followed up on at the surgery clinic where she had a benign abdominal examination but reported symptoms of gastric reflux and taking omeprazole three times daily. She had no bloody bowel movements or intermittent nausea. At this time, she was advised to follow up with her gastroenterologist and to take her proton-pump inhibitor only once daily for reflux.

## Discussion

According to the American Society for Gastrointestinal Endoscopy (ASGE) management of ingested foreign bodies and food impactions guidelines, in adults, true foreign body ingestion (i.e., nonfood objects) occurs more commonly in those with psychiatric disorders, developmental delay, and alcohol intoxication and those incarcerated individuals seeking secondary gain via release to a medical facility [2].

Ingestion of multiple foreign objects and repeated episodes of ingestion are common. Elderly adults are also at greater risk of ingesting foreign bodies, including an obstructing food bolus or dental prosthesis [2]. Patients presenting with food bolus impaction often have underlying esophageal pathology directly causing the impaction [2].

Impaction, perforation, or obstruction often occurs at GIT angulations or narrowing. Hence, patients with previous GI tract surgery or congenital gut malformations are at increased risk [2]. Once through the esophagus, most foreign bodies, including sharp objects, pass uneventfully. However, ingestion of sharp and pointed objects, animal or fishbones, bread bag clips, magnets, and medication blister packs increases the risk of perforation [2].

Clinical diagnosis of foreign body ingestion requires the use of multiple modalities such as a detailed history, physical exam, radiographic evaluation, and endoscopic evaluation as needed [3]. Older children and non-impaired adults may explicitly report ingestion of foreign bodies and localize discomfort in the abdomen, but these are frequently not correlated with the site of impaction or perforation and typically occur long after the patient ingests the foreign body [2].

Plain radiography poorly visualizes fishbones in soft tissues, with visibility varying by fish species, and the location and orientation of the bone [4]. Fishbones are difficult to visualize on plain radiographs with low sensitivity ranging from 25% to 39% but high specificity up to 91% when visible [4]. Clinical presentation and plain radiography are unreliable in the preoperative diagnosis of fishbone perforation of the GIT [4]. CT scan is the test of choice to radiographically diagnose fishbone impactions and is consistently accurate in revealing the offending fishbone with a sensitivity and specificity of 100% [5,6]. From the most radiodense to the least dense on CT scan, the different fish species range from bass, catfish, drum tilapia, salmon, trout, and red snapper to tilefish [4]. A CT scan can provide the clinician with information about the location, its relation with the adjacent structure, the presence of an abscess, and the signs of inflammatory processes [7]. CT scan also helps in the design of the type of management [6].

According to the guidelines of ASGE, foreign body ingestion with persistent esophageal symptoms should be evaluated by endoscopy even with a negative radiographic evaluation [2]. Sharp-pointed objects which have failed to pass should be followed with daily radiographs to document their passage, and surgical intervention should be considered for objects that fail to progress after three days [2]. Also, the patient should be instructed to report abdominal pain, vomiting, persistent temperature elevation, hematemesis, or melena. In the case of small-bowel foreign objects, a single- or double-balloon enteroscopy can be used for the retrieval of retained objects with the potential to cause obstruction or perforation; however, there is currently limited data validating the use of balloon enteroscopy for extraction of foreign bodies [2]. The decision has to be made based on the patient's clinical stability, availability of accessories (such as baskets, hoods, and forceps), length of procedure, and whether an antegrade or retrograde approach is preferred [2].

Surgical management is indicated in cases of peritoneal signs secondary to perforation, abscess formation, blood vessel penetration, severe inflammation, or bleeding [4,8]. Nonsurgical management of bowel perforation depends on the size and the location of perforation, diagnosis time, patient condition, and degree of contamination [4]. Some anatomical locations provide containment of the contamination such as the retroperitoneum and omentum; other locations necessitate surgical management, such as intraperitoneal, unless it is determined to be a micro-perforation [4]. Of note, in a retrospective study performed by Goh et al., 62 patients underwent surgery for ingested foreign body perforations, and 93% of these patients had ingested toothpicks or dietary foreign bodies such as fishbones or bone fragments [9]. Most of the perforations (70%) were intraabdominal, most commonly at the distal ileum (39%), while 29% of perforations occurred at the distal rectum or anus [9]. The study also noted that most foreign body perforations at the stomach, duodenum, or large intestines were more likely to be afebrile, have chronic symptoms (3 days or more), have a normal WBC count, and to be asymptomatic or present with abdominal mass or abscess compared to foreign bodies in the jejunum or ileum [9]. In our patient's case, the fishbones were found to be impacted in the greater and lesser curvatures of the stomach, jejunum, and ascending colon. We also suspected that the jejunal fishbone caused a micro-perforation and migrated into the adjacent mesentery during this patient’s initial admission.

Nonsurgical management should also include nutritional support, intravenous fluid, broad spectrum antibiotics, controlling the source of contamination, and organ support [6]. There is no specific duration for antibiotics, some physicians use it for 7-14 days, while others depend on the WBC level or clinical picture; nowadays, it's used for 5-7 days if patients have improved clinically [6].

In our patient’s case, none of the aforementioned surgical indications were present throughout their inpatient admission despite radiographic evidence of one fishbone remaining within the mesenteric fat due to a suspected micro-perforation of the jejunum. Subsequent discussions between the surgical, gastroenterology teams, and the patient revolving around future needs for surgery or possible complications. A consensus was reached, and all parties involved agreed to pursue nonsurgical management which included endoscopic and colonoscopic removal of the fishbones, supportive treatment with intravenous fluids, antibiotics, and bowel rest. This choice of treatment was related to the probable micro-perforation of the fishbone in the LUQ and then subsequent movement of this fishbone into the mesentery with no accompanying pneumoperitoneum. This choice was also considered due to the possibility of the fishbone being displaced if surgical intervention was attempted; it would increase surgery-related risks in an otherwise asymptomatic patient. Subsequently, the patient agreed to pursue outpatient follow-up and was advised on return precautions in the event she developed abdominal pain, vomiting, obstipation, persistent temperature elevation, hematemesis, melena, or rectal bleeding. Due to the complex nature of the patient's pathology, collaboration between a number of medical professionals was needed both while managing and writing this account.

## Conclusions

In conclusion, fishbone foreign body ingestion is a very common complaint. Although a variety of serious complications associated with gastrointestinal tract perforation may arise, they are uncommon. Diagnosis of perforation can be difficult in the setting of limited history highlighting explicit foreign body ingestion and overlapping clinical presentation of various abdominal pathologies, requiring clinically correlated physical examination and extensive radiographic evaluation with appropriate modalities such as CT scan. Subsequent management can be conservative or surgical depending on the patient’s clinical status, the location of the foreign body in the upper or lower gastrointestinal tract, and the presence or absence of complications such as peritoneal signs, sepsis, and radiographic identification of bowel perforation.
